# An Imaging and Computational Algorithm for Efficient Identification and Quantification of Neutrophil Extracellular Traps

**DOI:** 10.3390/cells11020191

**Published:** 2022-01-06

**Authors:** Apurwa Singhal, Shubhi Yadav, Tulika Chandra, Shrikant R. Mulay, Anil Nilkanth Gaikwad, Sachin Kumar

**Affiliations:** 1 Pharmacology Division, CSIR—Central Drug Research Institute, Lucknow 226021, India; apurwasinghal96@gmail.com (A.S.); 786shubhiyadav@gmail.com (S.Y.); shrikantrmulay@gmail.com (S.R.M.); anil_gaikwad@cdri.res.in (A.N.G.); 2 Transfusion Medicine Department, King George’s Medical University, Lucknow 226021, India; drtulikachandra@gmail.com; 3 Academy of Scientific and Innovative Research (AcSIR), Postal Staff College Area, Sector 19, Kamla Nehru Nagar, Ghaziabad 201002, India

**Keywords:** neutrophils, NETs, NETosis, apoptosis, cellomics, high content screening, inflammation

## Abstract

Neutrophil extracellular traps (NETs) are associated with multiple disease pathologies including sepsis, asthma, rheumatoid arthritis, cancer, systemic lupus erythematosus, acute respiratory distress syndrome, and COVID-19. NETs, being a disintegrated death form, suffered inconsistency in their identification, nomenclature, and quantifications that hindered therapeutic approaches using NETs as a target. Multiple strategies including microscopy, ELISA, immunoblotting, flow cytometry, and image-stream-based methods have exhibited drawbacks such as being subjective, non-specific, error-prone, and not being high throughput, and thus demand the development of innovative and efficient approaches for their analyses. Here, we established an imaging and computational algorithm using high content screening (HCS)—cellomics platform that aid in easy, rapid, and specific detection as well as analyses of NETs. This method employed membrane-permeable and impermeable DNA dyes in situ to identify NET-forming cells. Automated algorithm-driven single-cell analysis of change in nuclear morphology, increase in nuclear area, and change in intensities provided precise detection of NET-forming cells and eliminated user bias with other cell death modalities. Further combination with Annexin V staining in situ detected specific death pathway, e.g., apoptosis, and thus, discriminated between NETs, apoptosis, and necrosis. Our approach does not utilize fixation and permeabilization steps that disturb NETs, and thus, allows the time-dependent monitoring of NETs. Together, this specific imaging-based high throughput method for NETs analyses may provide a good platform for the discovery of potential inhibitors of NET formation and/or agents to modulate neutrophil death, e.g., NETosis-apoptosis switch, as an alternative strategy to enhance the resolution of inflammation.

## 1. Introduction

Neutrophils, the most abundant leukocytes, participate in immunity and inflammation through diverse mechanisms including phagocytosis, respiratory burst, and degranulation [1,2,3]. A distinct antimicrobial function, the release of neutrophil extracellular traps (NETs) was first time demonstrated in 2004 to combat the extracellular pathogens [4]. Significant progress has been made so far in the understanding of NETs including mechanisms of NET formation, their beneficial and detrimental roles in diverse conditions [2,5]. NETs are majorly released through a lytic form of neutrophil cell death, a process defined as NETosis that differs from apoptosis and necrosis [4]. Though neutrophils are also observed to release vital NETs that extrude DNA and leave intact live functional cytoplasts [6]. NETosis involves chromatin decondensation and its subsequent extrusion in combination with bound granule proteins into the extracellular environment [4]. These fibrous structures are composed of nuclear as well as mitochondrial DNA decorated with histones and proteases [2,4]. Diverse pathogens including *Candida albicans*, *Streptococcus* *pyrogens*, *Staphylococcus* *aureus*, *Escherichia* *coli*, HIV, and recently SARS-CoV-2 have been shown to induce NETosis [7,8,9,10,11]. Although NETs were initially identified as a mechanism to entrap and kill extracellular pathogens, but in recent years NETs have been associated with multiple inflammatory and autoimmune conditions including periodontitis, systemic lupus erythematosus (SLE), acute respiratory distress syndrome (ARDS), rheumatoid arthritis, thrombosis, and atherosclerosis [2,5,12]. Thus, identification of small molecules, drugs or inhibitors that can modulate the NETosis may provide therapeutic intervention in diseases associated with exuberated NET production.

NETs being fragile structures, hyper-responsive nature of neutrophils and application of different techniques for identification of NETs have led to inconsistency and controversies in the field over the past two decades [13,14,15]. Interestingly, in addition to diverse pathogens, NETs can be induced in-vitro via diverse inducers such as phorbol myristate acetate (PMA), ionomycin, lipopolysaccharides (LPS), interleukin-8 (IL-8), immune complexes, and others [7,16,17,18,19,20,21,22]. These different stimuli are dependent on the activation of distinct signaling pathways including ROS, citrullination, and calcium signaling for NET formation [8,23,24]. Nevertheless, multiple methodologies including immunofluorescence microscopy, flow cytometry, microfluidic device, and ELISA have been utilized for the investigations of NETs with definite limitations [8,25,26,27,28,29,30,31,32,33,34]. The common detection of NETs relies on identification of decondensed chromatin with DNA-binding dye and its co-localization with granular or histones proteins using conventional fluorescence microscopy. Although this approach provides the information of characteristic changes in the nucleus, granules, and extrusion upon NETosis [25,26], but exhibits limitations in being tedious, time taking, error-prone, subjective, and end point analysis. In addition, it is pertinent to note that the processing of samples can cause artifacts and remove breakable NETs, or cells that have undergone early death. Recently developed microscopy-based semi-automated NETs quantification methods are also labor-intensive and some parameters often fail to uniformly comply across a large set of images [26,27]. Another common approach is multi-well plate-based quantifications of externalized cell-free DNA in the supernatant using Pico green kit or in situ detection of DNA with membrane-impermeable dyes such as sytox green with tittered cell numbers. These methods fail to provide specific detection of NETs-associated DNA, as measured DNA can be a result of necrosis or late apoptosis of neutrophils and morphological changes remain ambiguous [8]. While immuno-blotting and ELISA provide low-throughput, end-point, expensive, and time-consuming readouts of NETs [28,29]. Consistently, cytometry-based approaches fail to detect externalized/fragile NETs as sample processing causes dismantling of NETs and fragile cells, although detect early-stage of NETosis with nuclear swelling [30,31,32]. Together, this suggests the need for further standardization of experimental approaches to allow unbiased computational detection of NETs for consistent data across the laboratories and avoid inconsistencies in the field.

Here, we described high content screening–HCS Cellomics™-based identification and quantification of NETs formation. HCS Cellomics platform utilizes a combination of high-throughput live in situ cell imaging and computational algorithm for data analyses that is useful for various functional analyses [35,36]. This approach has been utilized to quantify bacterial phagocytosis in conjugation with labeled fluorescent beads or bacteria [37]. Here, we highlighted the importance of live cell imaging-based detection of NETs and further utilization of computational algorithm analyses for high throughput screening of diverse novel pharmacophores for modulation of NETosis. Membrane integrity and nuclear area-dependent approach allowed the quantification of NET-forming neutrophils at multiple time points with distinction from other deaths including apoptosis and necrosis.

## 2. Materials and Methods

### 2.1. Isolation of Human Neutrophils

The human peripheral blood from healthy volunteers was collected in EDTA containing tubes. Donors were free from any medical condition and any medication for the prior 72 h phlebotomy. The procedure was performed as per guidelines approved by the institutional ethics committee CDRI/IEC/2020/L14. Human neutrophils (polymorphonuclear leukocytes; PMNs) were isolated from blood upon centrifugation at 500 rcf and the buffy coat was collected. Red blood cells contamination was reduced by Dextran sedimentation and leukocyte enriched supernatant fraction was collected. Neutrophils were separated using discontinuous density gradients of Histopaque-1119 and 62% Percoll at 800 rcf for 25 min at low acceleration and decelerations. The layer enriched in neutrophils was collected from the interface and washed using centrifugation. More than 98% pure human neutrophils were suspended in RPMI and counted for further experiments.

### 2.2. Induction of NET Formation

Neutrophils (3 × 10^4^ cells required per well) suspended in RPMI Lonza containing 0.8 mM divalent cations, 1% FBS and 2 mM HEPES were labeled for 20 min with Hoechst-33342 (1 µg/mL) dye that stains all the cells and Sytox Green (100 nM), a cell-impermeable DNA dye to visualize NETs. Cells were added to corning black-walled, clear-bottom 96-well microplate at density of 3 × 10^4^ cells in 100 µL volume per well. For pathway analysis, inhibitors were added to RPMI medium containing 1% FBS and 2 mM HEPES prior to the addition of the cells to control the timing of drug treatment. Cells were added to plate well containing tested compound, vehicle or known inhibitors of NETosis including 10 μM DPI and 100 μM Cl-amidine both obtained from Cayman chemical, Michigan, USA, and incubated for 30 minutes. NETs were induced in neutrophils by the addition of 1–5 µg/mL ionomycin (Cayman chemical, Ann Arbor, MI, USA) and 20–200 nM PMA (Sigma-Aldrich, MA, USA) at 37 degrees and 5% CO_2_ for 1–34 h. NETs were analyzed using HCS- Cellomics as described below. For studies detecting apoptotic cell deaths, neutrophils were incubated with 1:50 dilution of Annexin V-APC (BD Biosciences, California, USA), in combination with Hoechst-33342 and Propidium Iodide (1 µg/mL). Staurosporine (Cayman chemical, USA) at 1 μM for 3 h was used as positive control for the induction of apoptosis in neutrophils.

### 2.3. HCS Studio-Based Acquisition of Images and Identification of NETs

**Figure 1:**
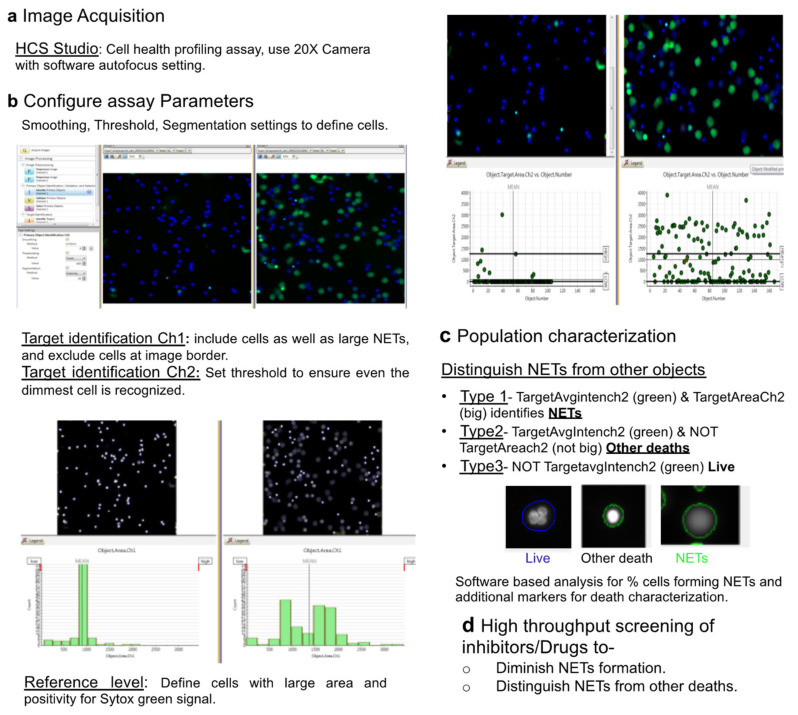
Representation of HCS Cellomics approach-based acquisition and specific detection of NETs. (**a**) HCS studio and cell health bioapplication-dependent image acquisition using 20× objective after Hoechst 33342-based autofocus of cells. (**b**) Configure assay parameters for visualization of NETs at single cell level. Identify primary objects using smoothing, threshold, and segmentation in channel 1 and channel 2. Revalidate the primary objects data after visualization of cells in the green and blue channel with the change in cell area and signal intensities and threshold. Select reference levels to differentiate between live, NET forming, and cells with compromised membrane integrity and no nuclear decondensation. (**c**) Identify distinct populations using differential Sytox average intensity Ch2 and Target Area Ch2 for different sets of parameters. Confirmed the results using back analysis of cell under investigation with particular area and intensity. (**d**) Apply this approach for high throughput screening of inhibitors/drugs as mitigator of NETs formation and/or switching to other deaths.

A 96-well plate was loaded and imaged with a 20× objective at HCS Cellomics™ (Thermo- Fisher Scientific ArrayScan™ VTI) system. Bioapplication Cell Health Profiling was used with modifications for the acquisition and identification of NETs. Cells stained with Hoechst-33342 and Sytox green were captured using software-based autofocus of Hoechst 33432 channel. High-resolution images of 6 to 10 fields/well were acquired using blue (386-23_BGRFRN_BGRFRN)) and green (485-20_BGRFRN_BGRFRN) channels in the HCS Studio 2.0 Software using a range of distinct settings for population characterization including autofocus, threshold, and segmentation (Figure 1). For Annexin V-APC and PI detection, far-red (650-20_BGRFRN_BGRFRN) and red (549-20_BGRFRN_BGRFRN) channels were used in the HCS Studio 2.0 Software. For time-dependent NETs analysis, images were acquired before (t = 0) and after addition of 100 nM PMA at every 15 min for 3 h with 20× objective using HCS Cellomics™. High-resolution images were captured using blue (386-23_BGRFRN_BGRFRN), red (549-20_BGRFRN_BGRFRN), and far-red (650-20_BGRFRN_BGRFRN) channels. For other studies, NETs were assessed at different time points based on the inducer used for NET generation. The percent NETosis was determined using HCS studio software as Sytox green or PI positive NET forming cells with an enlarged decondensed nucleus vs. total number of Hoechst positive cells.

### 2.4. Automated Image Analysis and Quantification of NETs

Neutrophils and NETs were identified using algorithm-based analysis of HCS studio software version 6.6.0 (Figure 1). The computational image analysis involved image preprocessing, object identification, validation, and selection for recognition and quantification of NETs. Image preprocessing was performed via background correction and smoothing to make objects more distinct and define edges of the cells. The primary object identification Ch1 (Hoescht stained nuclei) was performed with a processing definition set to recognize all the neutrophils per field of view via changing parameters like threshold and segmentation. Cells undergoing death, including apoptosis and NETosis, were identified with changes in cell permeability and distinct nuclear areas with Sytox green staining. The value of the threshold was kept to a value that allowed identification of even the dimmest cell/object so that all the cells in a field are included during analysis. Adjustment of segmentation value was used to resolve and identify individual cells. Primary objects were validated and target Ch2 mask modification was performed to define region of interest where measurements are to be made. Then, reference level was adjusted to identify different subpopulation of cells including NETs, live cells, and other death forms. Task settings Object Target.Area.Ch 2 (Sytox green) allowed us to define ‘’responder cells’’ with larger surface area, while Object Target.Avg.intensity Ch2 enabled to select minimum intensity that decides a green signal. Further, population characterization was performed to define cells with large surface area in green channel as NET-forming cells. Conversely, cells with small surface area and increased Sytox green intensity were defined as cells that had undergone death not through NETs. This allowed us to differentiate NET forming cells from other forms of cell deaths. Cells were identified with three distinct characters:

- Type 1 events, i.e., NET forming cells were identified using Target.AvgIntensity.Ch2 AND Target.Area.Ch2 as positive for green and big decondensed nucleus.
- Type 2 events, i.e., green and small cells that were undergoing death through pathways other than NETs were identified using Target.AvgIntensity.Ch2 NOT Target.AreaCh2. parameter.
- Type 3 objects were identified as NOT Target.AvgIntensity.Ch2 for live cells.

Once event definition was set, scan was performed. Launch view was used to view graph resulting number of objects and percentage of type, type 2, and type 3 objects representing the NET forming, other deaths, and live cells, respectively. Together, key steps for the specific detection and quantification of NETs are summarized in Figure 1.

### 2.5. Immunofluorescence Microscopy

Cells or NETs were fixed with 2% paraformaldehyde solution and labeled with granular protein and nuclear dye. Myeloperoxidase (MPO), a primary granule protein, was labeled using primary antibody against MPO overnight at 4 degrees followed by secondary Alexa Fluor 488 anti-rabbit antibody for 1 h at room temperature (Both obtained from Invitrogen, Waltham, MA, USA). Further nuclei were stained using DAPI containing mounting media. Only secondary antibody was used as specificity control. The co-localization of MPO and DNA was used for the identification of NETs. Images were captured and analyzed at 40× objective using 2 filters- DAPI, FITC on a Leica DMI6000 inverted fluorescent microscope.

### 2.6. Statistics

All the experiments were performed at least three times. Data are presented as mean ± SEM. An unpaired *t* test (normally distributed) was performed as statistics for comparison of experimental groups unless specified. To compare multiple groups, one-way ANOVA was used. The *p*-value of *, *p* < 0.05; **, *p* < 0.01; and ***, *p* < 0.001 were considered as significant.

## 3. Results

### 3.1. Validation of HCS-Cellomics Algorithm Using PMA Induced NET Formation

**Figure 2:**
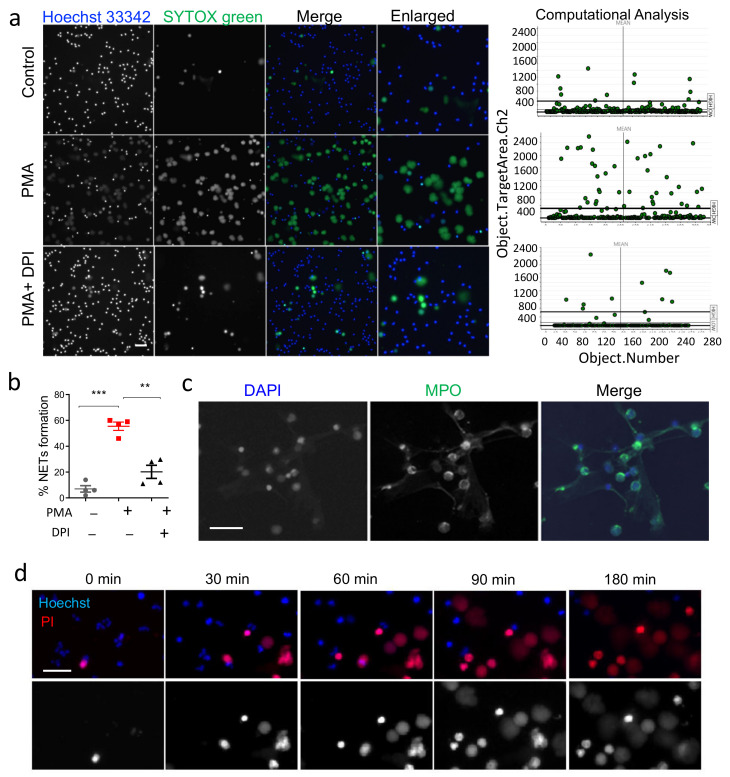
Analysis of PMA-induced NETs using HCS studio software and its inhibition by DPI. (**a**) Left side, representative images of control and neutrophils stimulated with 100 nM PMA and 10 μM of DPI at 3 h. The cells were identified using Hoechst 33342, a cell permeable nuclear dye that labeled nucleus in all cells. NETs were identified as positive for cell impermeable Sytox green and enlarged cells, scale bar; 50 μm. On right side, scattered plots represent HCS-based computational analysis of NETosis using object area channel 2. The percentage of NET forming cells were calculated as Sytox positive nuclei out of total Hoechst positive cells. (**b**) The effect of 10 μM DPI on PMA stimulated NETs, percentage of neutrophils. (**c**) Representative immuno-staining pictures of PMA-stimulated neutrophils at similar condition confirming validity of HCS-based measurements. (**d**) Time lapse analysis of PMA-induced neutrophils using Hoechst 33342 and PI dyes. ** *p* < 0.01, *** *p* < 0.001; Data are means ± SE.

PMA is one of the most extensively utilized inducers of NETs with a known mechanism of action [4,8,26]. To aid easy, rapid, and specific detection of NETs, we first developed a HCS-Cellomics-based platform to identify and quantify NET formation. NET formation was induced with PMA (20, 100, and 200 nM) and was evaluated using Sytox green and Hoechst dyes on the HCS Cellomics™ system. After autofocus, 6–10 fields/well were acquired, data were captured and analyzed using HCS Studio Software based automated image analysis (Figure 1). We observed that, PMA at 100 nM provided consistent induction of NETs. Neutrophils in RPMI media containing 1% FBS and 2 mM HEPES were considered as a control and did not show any significant change in the viability of unstimulated human neutrophils for the study period up to 4 h (Figure 2). HCS Cellomics system recorded good quality images and HCS studio software analysis led to specific identification, and quick quantification of NET forming enlarged Sytox positive cells (Figure 2a). NOX inhibitor, DPI at 10 μM mediated inhibition of PMA induced NET formation in the present system validated the role of NADPH oxidase-dependent ROS pathway (Figure 2b), a phenomenon well reported previously [8]. The data observed using HCS Cellomics were further validated using fluorescent microscopy at similar conditions. PMA induced NETs stained with nuclear dye, DAPI were co-localized with granular protein, MPO (Figure 2c). This approach was similarly effective with another cell-impermeable DNA dye, Propidium Iodide, and also provided time-dependent changes in PMA-induced NET formation and their good quality image analysis (Figure 2d). Together, HCS Studio Software-based computational algorithm provided reliable high throughput analyses of NETs.

### 3.2. Validation of HCS-Cellomics Algorithm Using Ionomycin-Induced NET Formation

**Figure 3:**
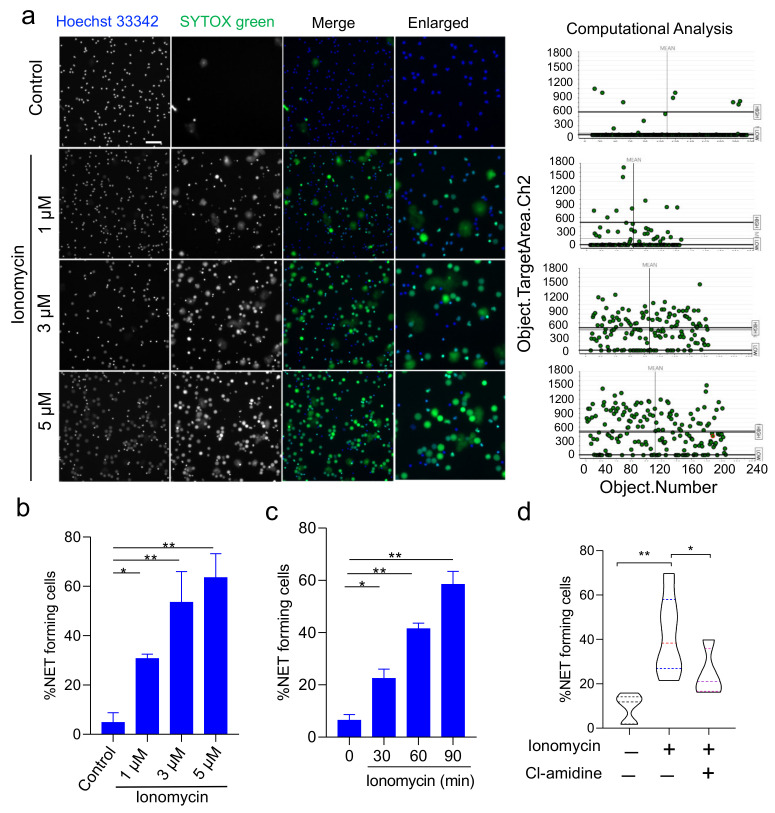
Ionomycin-induced NETs and HCS Cellomics-based analysis. (**a**) Left side, representative images of control, Ionomycin-treated neutrophils acquired at 20× objective. The cells were treated with different concentrations of ionomycin for 90 min as indicated in the figure and analyzed based on Hoechst 33342 and Sytox green staining with decondensation of nucleus. Scale bar; 50 μm. Right side scattered plots represent target area in channel 2 (sytox green channel) used for identification of cells forming NETs with enlarged decondensed nucleus. (**b**) The concentration and (**c**) time-dependent effect of Ionomycin on NET formation from human neutrophils. (**d**) Effect of 100 μM PAD inhibitor Cl-amidine on Ionomycin-induced NETs. * *p* < 0.05, ** *p* < 0.01; Data are means ± SE.

To further validate HCS-Cellomics-based analysis of NETs, we utilized another widely used NETs stimulator, a calcium ionophore- ionomycin. Interestingly, ionomycin induces NETs in an NADPH-independent manner, through calcium signaling [38]. The concentration dependent effect of ionomycin at 1–5 µM was evaluated. We found an increase in the percentage of NET forming cells with a simultaneous decrease in live cells upon increasing concentration of ionomycin treatment (Figure 3a,b). Consistently, HCS-Cellomics-based high-throughput live in situ cell imaging provided time-dependent increase in NETs upon ionomycin treatments (Figure 3c). Our assay also confirmed the role of protein arginine deiminase 4 (PAD4) as 100 μM Cl-amidine inhibited ionomycin-induced NETs significantly (Figure 3d), emphasizing its utility as a novel research tool to study NETs formation. Together, HCS Studio Software-based computational algorithm provided reliable high throughput analyses of NETs.

### 3.3. HCS-Cellomics Distinguishes NETs from other Forms of Neutrophil Death

**Figure 4:**
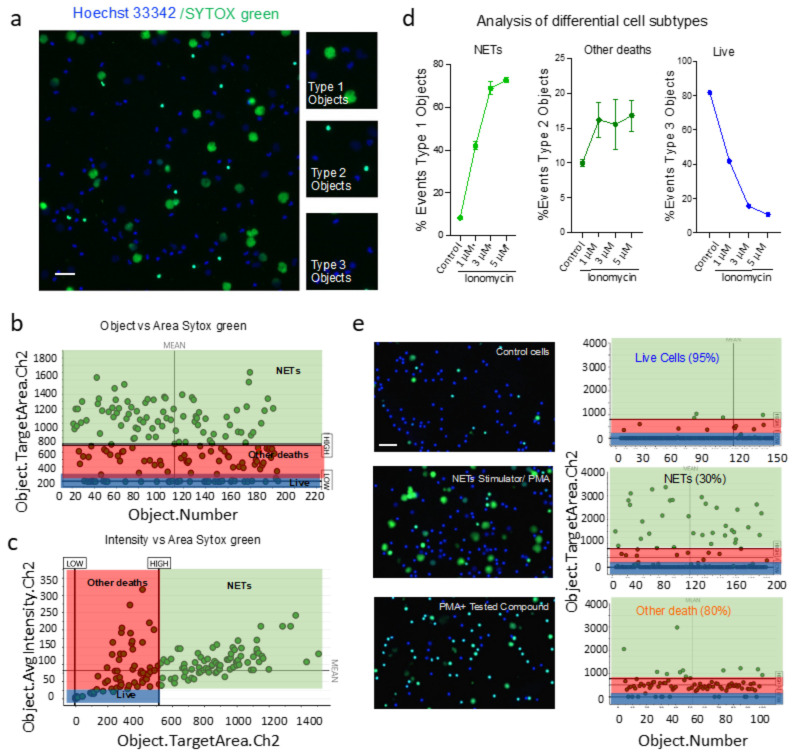
HCS-Cellomics analyses discriminate NETs from other death pathways. (**a**) Representative image and Ch2 target area analysis of neutrophils treated with 1.5 μM Ionomycin. Inserts define distinct cell types observed. Type 1 events, i.e., NET forming cells were positive for green and big decondensed nucleus, Type 2 events, i.e., green and small cells that were undergoing death through pathways other than NETs. Type 3 objects as live cells. Scale bar; 50 μm. (**b**,**c**) Target area channel 2 analysis and its comparison to target mean intensity channel 2 provides discrimination between live, dead, and NET forming cells. (**d**) Quantification of cells exhibiting NET formation, cell undergoing distinct death or live cells. (**e**) Images of neutrophils undergoing NETs and other death forms in response to 10 μM of tested compound, Dot plot analysis of Target area channel 2-differentiated NET forming cells and other death forms.

NET formation often involves death of neutrophils [4]; however, there is no consensus on the underlying mechanisms of cell death. Here, we employed HCS-Cellomics to distinguish NETs from other forms of cell death. Briefly, the nuclear area, fluorescence intensities, and other analyses were recorded for specific detection of NET forming cells in the assay described above. Data are represented as the percentage of neutrophils forming NETs, as well as live cells or cells undergoing other form of deaths. NETs are decondensed enlarged nuclear structures that depict positivity for Sytox green dye upon disintegration of membrane. Interestingly, we observed a fraction of cells with Sytox green staining, but intact/condensed nuclei in PMA and ionomycin-stimulated cells. These features suggested compromised membrane integrity and a death distinct from NETosis. Vigilant analysis of these groups using HCS Studio software led to the identification of three distinct types of cells in stimulated groups. Type 1 events, i.e., NET forming cells were positive for Sytox green and exhibited enlarged decondensed nucleus, Type 2 events, i.e., Sytox green positive, but smaller cells that most likely undergone death through different pathways other than NETs. Type 3 objects do not stain for Sytox green as live cells (Figure 4A). These populations can be differentiated using Sytox green area analysis alone or in combination of Sytox green mean intensity analysis (Figure 4B,C). It is indeed very important to differentiate these populations, as low percentage of these cells, i.e., dying but not through NETosis pathway were also present in unstimulated group and further enhanced upon ionomycin treatment (Figure 4D). Furthermore, novel unknown potential inhibitors may drastically increase these type 2 cells, a therapeutic option in a context-dependent manner [39,40]. To validate the application, we tested our high throughput algorithm assay on 96-well plates, which resulted in differential changes in NET-forming cells and type 2 cells (other deaths) upon treatment with different inhibitors or FDA approved drugs. For example, we found a profound increase in the type 2 objects upon stimulation with PMA in the presence of a tested compound (Figure 4E). Together, HCS computational algorithm was able to identify NETs and distinguish them from other death forms induced that were validated using image visualization. Thus, HCS-Cellomics distinguishes NETs from other forms of neutrophil death.

### 3.4. Analysis of NETs, Apoptosis, and Necrosis with HCS-Cellomics and Flow-Cytometry

**Figure 5:**
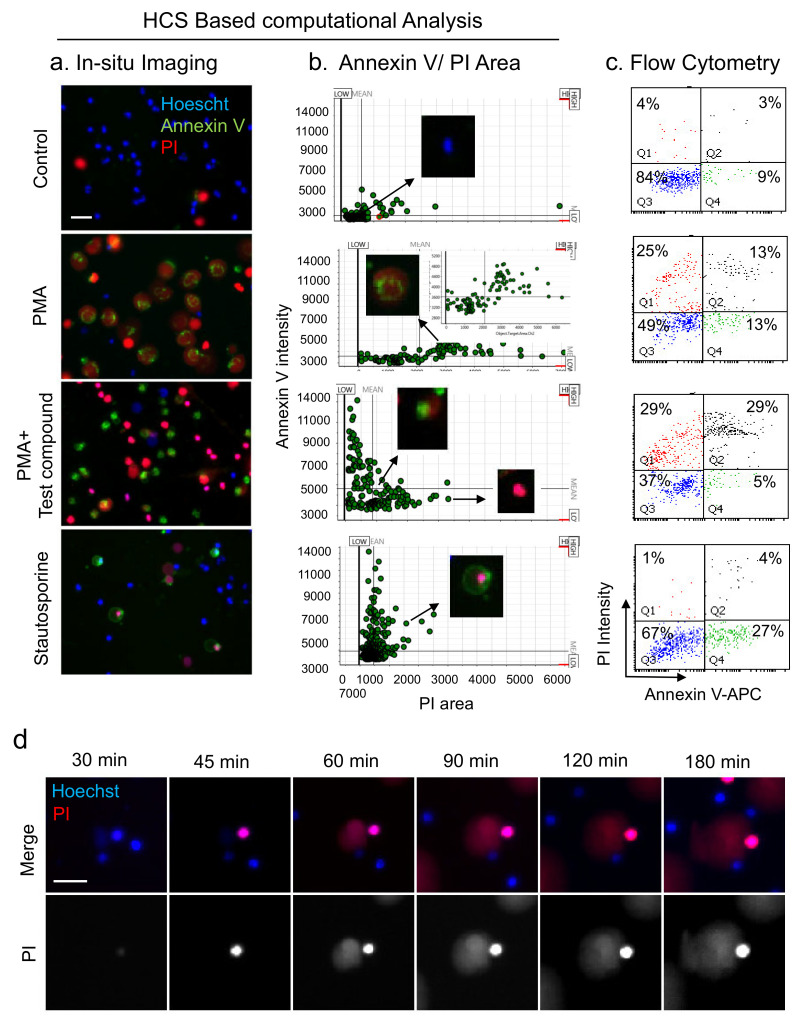
HCS-Cellomics and flow-cytometry-based analyses of NETs, apoptosis, and necrosis. (**a**) Representative image of Annexin V, PI, and Hoescht positive cells in different treatment groups. Cells were treated with 10 μM tested compound, 100 nM PMA, or 1 μM staurosporine for 3 h and analyzed for distinct cell types using HCS Cellomics. NET forming cells were identified as PI (red) positive cells with large decondensed nucleus and punctate Annexin V staining. Necrotic cells, i.e., red and small cells that were undergoing death through pathways other than NETs. Staurosporine-induced apoptotic cells were marked with surface localized Annexin V staining and condensed PI positive nuclei in contrast to NETs. (**b**) Dot plot analysis of PI area and its comparison to Annexin V mean intensity provides discrimination between live, NET forming cells, and other death form, necrosis. (**c**) Flow cytometric analysis of live cells and distinct death forms in the presence of different treatments mentioned in panel A, using Annexin V/PI staining. (**d**) Time lapse tracing of neutrophils undergoing distinct death forms, specifically necrotic cell with early permeability and condensed nucleus identified using PI staining, while NETs represented late loss of permeability, with decondensed nuclear structure.

The condensed nucleus with compromised membrane integrity in PMA-treated cells in presence of tested compound (Figure 4E), mimicking late phase apoptosis instigated us to investigate Annexin V staining in different sets of treatments. During apoptosis, Annexin V binds to PS externalized to the outer membrane from inner leaflet of the cell membrane as a result of flippase activity during early apoptosis without loss of membrane permeability. Similarly, Annexin V remains positive in the late phase of apoptosis characterized with the loss of membrane integrity [41]. Interestingly, Annexin V can also bind to intracellular phosphatidyl serine revealed after rupture of cells during NETosis as well as necrosis, and thus fails to differentiate late apoptotic cells from NET forming cells [8,41]. Thus, we evaluated differential changes in membrane permeability, nuclear area, and annexin V staining in different groups including PMA-induced NETs, Staurosporine dependent apoptosis, and changes with tested compound (Figure 5). Control cells were majorly alive, while treatment with 1 μM staurosporine-induced apoptosis in neutrophils (Figure 5a,b). Computational analysis revealed a substantial increase in type 2 objects including necrotic (only PI positive, not NETs) and apoptotic cells (Annexin V positive, not NETs) in the presence of test compound in PMA-stimulated cells (Figure 5a,b). While PMA-induced NETs, identified using enlarge decondensed nuclear area, also exhibited disintegrated annexin V staining most likely at compromised plasma membrane results of NETs release (Figure 5a,b and insert) [8]. We also performed flow cytometry analysis using annexin V and PI staining, a phenomenon well reported in literature for NETosis as well as apoptosis [31]. Consistent with HCS data, flow-cytometry analysis revealed presence of annexin V and PI positive cells in PMA-treated NETosis group, but failed to distinguish them from late apoptotic cells (Figure 5c). Moreover, sample processing for FACS analysis likely breaks fragile cells/NETs, thus provided an increase in nuclei positive only for PI (Figure 5c), which is distinct from HCS-based live cell imaging-based Annexin V positive, enlarged, decondensed NETs in PMA and tested compound A groups (Figure 5a,b). Further, time-lapse analysis of neutrophils upon exposure to 100 nM of PMA, led to identification of possible transition and interlink of distinct deaths. We observed induction of type 1 (NETs) and type 2 death forms in neutrophils. Interestingly, necrotic (only PI positive, not NETs) death get induced around 30 min time point and that did not show any DNA extrusion even after prolonged incubation (Figure 5d). In contrast, NETs forming cells exhibited marked nuclear decondensation with increase in the PI surface area over time. This different sequence of events suggests entirely distinct cell death programs in NETosis and necrotic death forms (Figure 5d). Together, this study also highlights the importance of a combination of in situ high-throughput live cell imaging without any alteration in cells and NET structures during sample processing, including making suspension (flow cytometry), fixation, and permeabilization (immunofluorescence microscopy). This approach can also identify a putative switch of NETosis to apoptosis with distinct drug molecules, a desirable approach for early resolution of inflammation, with non-inflammatory clearance of apoptotic neutrophils. Altogether, HCS provides an efficient quantitative method to differentiate NET-forming cells from other death forms, including apoptosis, necrosis, and overcomes major existing limitation with other protocols.

## 4. Discussion

Due to NETs releasing ability, neutrophils function as “atomic bombs” of the immune system [42]. These NETs enriched in DNA, proteases, oxidants, and MMPs can cause damage to different cells, including lung cells, and further enhance cytokine storm syndrome. Moreover, the severity and mortality of ARDS patients are directly linked to NETs [43,44]. Indeed, a recent study identified exuberated NETs in broncho-alveolar lavage and lung tissue identified as cell-free DNA, MPO-DNA complex in COVID-19 patients [11]. Therefore, NETosis has been acknowledged as a therapeutic target for reducing exposure of toxic oxidants/proteases in broncho-alveolar tissue and subsequent cytokine storm, microvascular thrombosis, coagulopathy, and ARDS [45]. However, lack of specific and quantitative computational approaches for analyses of NETs has hindered explicit therapeutics targeting of NETs formation.

Approaches like immunofluorescence microscopy and ELISA-based detection of externalized DNA in combination with granular proteases are prone to bias and lack of information related to early time points during the NET formation process [25,26]. Sytox dyes have been extensively utilized to visualize NETs based on its non-permeable nature and non-fluorescence without its interaction with DNA that make them good for detecting NETs in-situ without washing of unbound dye. However, it still can provide false positives due to apoptosis or necrosis of neutrophils and susceptibility to cationic peptides [46], a major disadvantage for Sytox fluorescence measurement using fluorometry/plate reader that require supplement microscopic analysis with granular proteins or histones [46]. Though, cytometry-based detection methods of NETs are specific, but also highly sensitive to washing procedures during sample processing that might be responsible for a low percentage of NET forming cells observed utilizing these methods in contrast to microscopy-based approaches [8,32]. Flow cytometric processing of samples results in fragmentation of fragile cells/NETs and leads to change in size and granularity of cells, NETs, with loss of enlarged decondensed NETs forming cells. Additionally, a consistent strategy is not used amongst the researchers for the identification of NETs [30,31,47]. In this study, HCS Cellomics using high-throughput cell imaging and computational algorithm dependent data analyses dissected out of different deaths without interference from the above-mentioned problems.

Using image-stream analysis, Zhao et al. argued to identify suicidal and vital NETs, but failed to show live functional anuclear cells [32]. Conversely, elongated cells were observed with intact condense nucleus and distinct granules, which are hallmarks of cell polarization during chemotaxis with different stimuli [48]. Image-stream analysis is associated with sample processing and likely breakage of fragile cells as well as NETs. This suggests requirements of methodologies with minimal disturbance of NETs and automated quantification. On this note, IncuCyte ZOOM platform in a recent study distinguished various neutrophil cell deaths with morphological changes in the cell and nucleus [25], but this platform lacks potential for high throughput analyses. Our study provides an optimized method for detection and unbiased analyses of NETs and additionally offers cell polarization characteristics of live functional nuclear cells for example with Calcein-am dye (not shown). Moreover, our HCS-Cellomics algorithm also allows time-lapse analysis. Chicca et al. [49] utilized HCS platform on fixed cells using single channel hoechst-33342 or Sytox green. Interestingly, fixation of cells permits the internalization of Sytox green into cells, which defies utility of cell-impermeable DNA dye, for discrimination of NETs from other deaths and live neutrophils. On the other hand, our study analyzed the NET formation in a temporal and longitudinal manner and also distinguished NETosis from other cell death forms based on negative staining for cell-impermeable nuclear dye and nuclear area analysis.

The low throughput, tedious, high cost, and user bias of microscopy led to several recent developments of computational approaches for quantifying NETs [50]. For example, Coelho et al. utilized a supervised algorithm on visually annotated images, this regression model utilizes the knowledge of Python [51]. Interestingly, the MATLAB application NETQUANT utilizes cell surface area, DNA deformability, NET bound protein level to quantify NETs [52]. Still, these computational analyses require NETs images, and thus, lack the real high throughput application, speed, un-biasness, and optimization for variation in NETs driven from diverse stimuli [24]. Hoffmann et al. [53] demonstrated inter-individual variation in the predisposition of neutrophils towards NETosis, consistently we also observed low or high NETosis prevalence in blood isolated from different healthy donors [53]. In the present study, inter-individual variations were normalized and analyzed using positive stimulator, and known inhibitors as proof of concept. Moreover, backtracking NET event at the single-cell level helps in minimizing artifacts with optimum segmentation, thresholding, and thus might provide reproducible data across laboratories.

HCS-Cellomics comprises fluorescence microscopy, image processing, automated cellular measurements, image analysis algorithm, and informatics tools, provides a powerful platform for computational unbiased, quantitative and reproducible analyses of NETs in high throughput manner. This further differentiates NET-forming cells from apoptotic or necrotic cells, a major limitation existing with other methodologies. Though we utilized this for NETs analyses under in-vitro setting, it may be well adapted for analysis of neutrophil extracted from inflammatory tissues. Mechanistic inhibitors of NETs associated pathways like PAD4, ROS, MPO, CXCR2 are of potent hope towards targeting NETs and neutrophil associated inflammation in diseases [54], but remain far from clinical use so far. Intriguingly, our imaging and computational algorithm-based high throughput screening may help to identify novel small molecules, FDA approved drugs, and natural products to therapeutically target NETs. Interestingly, in addition to mitigation of pro-inflammatory NETs, switching to apoptotic death is highly desirable due to its non-inflammatory nature [45]. Together, this approach provides an efficient, specific, and high throughput investigation of NETs and screening of specific drugs for NETs-associated clinical pathologies.

## 5. Conclusions

Neutrophil extracellular traps are associated with various disease conditions and have been a focus of research. Nevertheless, limitations of various methodologies used for detection and quantifications of NETs led to inconsistencies. This method using HCS-Cellomics-based algorithm utilizes high-throughput cell imaging in combination with data analyses and differentiates NETs from apoptosis and necrosis. Our algorithm can be used to screen novel drugs and compounds to target NETosis and/or modulate the inflammatory neutrophil NETosis to apoptosis switch. Importantly, this approach can be efficiently used for other compatible and capable systems in addition to HCS-Cellomics.

## Data Availability

Data and material included in the manuscript, including relevant raw data, may be made available to any researchers for non-commercial purposes, while preserving any necessary confidentiality and anonymity.
